# The Impact of Movement Behaviors on Bone Health in Elderly with Adequate Nutritional Status: Compositional Data Analysis Depending on the Frailty Status

**DOI:** 10.3390/nu11030582

**Published:** 2019-03-09

**Authors:** Irene Rodríguez-Gómez, Asier Mañas, José Losa-Reyna, Leocadio Rodríguez-Mañas, Sebastien F.M. Chastin, Luis M. Alegre, Francisco J. García-García, Ignacio Ara

**Affiliations:** 1 GENUD Toledo Research Group, Universidad de Castilla-La Mancha, 45071 Toledo, Spain; Irene.rodriguez@uclm.es (I.R.-G.); asier.manas@uclm.es (A.M.); losa.jose@gmail.com (J.L.-R.); luis.alegre@uclm.es (L.M.A.); 2 CIBER of Frailty and Healthy Aging (CIBERFES), 28001 Madrid, Spain; leocadio.rodriguez@salud.madrid.org (L.R.-M.); franjogarcia@telefonica.net (F.J.G.-G.); 3 Geriatric Department, Hospital Virgen del Valle, 45071 Toledo, Spain; 4 Geriatric Department, Hospital Universitario de Getafe, 28901 Getafe, Spain; 5 School of Health and Life Sciences, Glasgow Caledonian University, Glasgow G1 1BX, UK; Sebastien.Chastin@gcu.ac.uk; 6 Department Movement and Sport Sciences, Ghent University, 9000 Ghent, Belgium

**Keywords:** bone mineral density, light physical activity, moderate-to-vigorous physical activity, sedentary time, aging

## Abstract

The aim of this study was to determine the relationship between bone mass (BM) and physical activity (PA) and sedentary behavior (SB) according to frailty status and sex using compositional data analysis. We analyzed 871 older people with an adequate nutritional status. Fried criteria were used to classify by frailty status. Time spent in SB, light intensity PA (LPA) and moderate-to-vigorous intensity PA (MVPA) was assessed from accelerometry for 7 days. BM was determined by dual-energy X-ray absorptiometry (DXA). The combined effect of PA and SB was significantly associated with BM in robust men and women (*p* ≤ 0.05). In relation to the other behaviors, SB was negatively associated with BM in robust men while BM was positively associated with SB and negatively with LPA and MVPA in robust women. Moreover, LPA also was positively associated with arm BM (*p* ≤ 0.01). Finally, in pre-frail women, BM was positively associated with MVPA. In our sample, to decrease SB could be a good strategy to improve BM in robust men. In contrast, in pre-frail women, MVPA may be an important factor to consider regarding bone health.

## 1. Introduction

The aging process brings several physiological changes such as a decrease of bone mass and osteoporosis, a skeletal disorder characterized by reduced bone mineral density (BMD) and mass, resulting in damaged bone structure [1]. According to this definition, people are categorized depending on the BMD into three groups based on dual-energy X-ray absorptiometry (DXA) T-score: normal [−1 standard deviation (SD) or over], osteopenia [−1 SD to −2.5 SD], osteoporosis [−2.5 SD or below] [1]. Bone health deterioration and osteoporosis can lead to a decrease in quality of life, disability, institutionalization, and mortality, making this disease an important contributor to the public health burden [2]. These bone health problems are closely related to frailty [3,4], which is considered a biological condition where there is poor resolution of several physiological systems to maintain homoeostasis after a low-power stressor event [5,6]. Osteoporosis and frailty share many common risk factors, such as malnutrition, sarcopenia, physical inactivity, and low vitamin D [7,8]. Therefore, previous studies have investigated the relationship between frailty and BMD and content (BMC) [3,4,9] with discrepant results and without specifically considering nutritional status. Nevertheless, it is very important to study each frailty status independently (robust, pre-frailty and frailty), due to the fact that the risk of osteoporosis and bone fractures is higher in pre-frail and frail individuals [10,11]. Likewise, sarcopenia, a syndrome characterized by progressive and generalized loss of skeletal muscle mass and strength with a risk of adverse outcomes such as physical disability, poor quality of life and death [12], can be considered as other of the main physical drivers of frailty, although frailty is not considered a component of sarcopenia [13]. The influence of sarcopenia on bone health and which can be explained from Harold Frosts Mechanostat Theory [14]. Thus, lifestyle and physical activity (PA) are considered one of “the golden rules” of osteoporosis treatment and as one of the keystones in the development of frailty [15,16,17]; although the optimal dose of exercise for bone health is yet to be fully determined in high-risk individuals [18]. Consequently, some recent research indicated the need to study how PA and sedentary behavior (SB) could simultaneously influence frailty and health variables [5,18,19]. Furthermore, as time within a 24-h period is finite, time spent in different movement behaviors is intrinsically collinear and co-dependent [20]; thus, time spent in one behavior necessarily reduces time spent in at least another one. To understand clearly the relation between PA and bone mass, it is necessary to use compositional analysis; a method that allows dealing directly with the fundamental nature of movement behavior data which are intrinsically compositional [20].

Therefore, our hypothesis is that the PA levels would be positively associated with bone health depending on the lifestyle and the frailty status. The present study was therefore designed (1) to determine the relationship between bone mass and the movement behaviors distribution according to frailty status and, (2) to examine whether differences in this distribution between older men and women with adequate nutritional status exist.

## 2. Materials and Methods 

### 2.1. Study Sample and Design 

This cross-sectional research selected subjects from the Toledo Study for Healthy Aging (TSHA), a Spanish population-based prospective cohort study. The full methodology was described previously [21]. To date, this study has completed three waves: 2006–2009, 2011–2013, and 2015–2018. In the present study cross-sectional data from subjects belonging to the second and third wave were included as DXA measurements and accelerometer data was only collected during these waves. Only the subjects with an adequate nutritional status according to the Mini-Nutritional Assessment questionnaire for screening of malnutrition risk were included [22]. Therefore, the sample was composed of 871 participants: 395 men (76.9 ± 5.3 years) and 476 women (76.7 ± 4.7 years). The study was approved by the clinical research ethics committee of the Toledo Hospital Complex (approval code: 2010/93) and all the subjects signed an informed consent to be included in the study.

### 2.2. Anthropometrics and Bone Health and Body Composition

Anthropometric measurements were obtained on each subject immediately before DXA assessment by a balance-stadiometer Seca 711 (Hamburg, Germany). BMC (g) and BMD (g/cm^−2^) were carried out by DXA (Hologic, Serie Discovery QDR densitometer, Bedford, MA, USA). Whole body and regional sites (arms and legs), lumbar spine (L1–L4), and the proximal region of the femur (femoral neck) were assessed in a supine position, wearing light clothing with no metal, shoes or jewelry. The arm region included the hand, forearm, and arm and was separated from the trunk by an inclined line crossing the scapulohumeral joint, such that the humeral head was located in the arm region. The leg region included the foot, the lower leg, and the upper leg. It was separated from the trunk by an inclined line passing just below the pelvis, which crossed the femoral neck. The bone T-scores were calculated in each participant for the femoral neck. Whole body fat mass (g), lean mass (bone-free) (g) and percentage body fat mass were also obtained from the total body scan. Sarcopenia was evaluated as stated by the specific criteria for our DXA model proposed by Delmonico et al. (2007) [23]. Physician’s Viewer, APEX System Software Version 3.1.2. (Bedford, MA, USA) was used to analyze all scans. DXA was calibrated daily against a lumbar spine phantom following the manufacturer’s guidelines.

### 2.3. Physical Activity and Sedentary Behaviors

PA and SB were measured using ActiTrainer and ActiGraph wGT3X-BT (ActiGraph, LLC, Pensacola, FL, USA) accelerometers. Participants were instructed to wear the accelerometer on the left hip during waking hours for seven consecutive days and remove it during any bathing or swimming activities. The study included only the results from participants with at least four valid days with at least 480 min (8 h) of wear without excessive counts each day (i.e., >20,000 counts), independent of whether it was weekdays or weekends, as in previous studies [24,25]. Data were extracted using 60 s epochs for the subsequent intensity analyses using ActiLife Pro 6 software. Consecutive strings of zero >60 min were defined as non-wear time and were removed [26]. Each valid wearing-time minute was classified into one of the classical intensity bands using count-based thresholds: SB (<1.5 METs), light physical activity (LPA) (1.5–2.99 METs) and moderate-to-vigorous physical activity (MVPA) (≥3 METs). Elderly-specific cut-off points for vector magnitude counts per minute were used in this analysis [27,28]. The values were normalized to total wear time and averaged over the number of valid days to derive an estimate of the mean time spent in SB and each physical activity level per day.

### 2.4. Frailty Status

The frailty syndrome was evaluated as stated by the criteria proposed by Fried et al. (2001) [6]. To analyze all data, we classified all subjects according to their frailty status as frail if they presented ≥3 criteria (unintentional weight loss, slowness, weakness, physical inactivity or fatigue), pre-frail in case of one or two criteria or robust if they did not have any of the criteria.

### 2.5. Data Analysis

Statistical analyses were completed using R statistical system version 3.1.1., following the guide to compositional data analysis for PA, SB, and sleep research published by Chastin and colleagues [20]. The characteristics of the study groups were determined through basic descriptive tests (mean ± SD) and compositional descriptive statistics (compositional geometric means for central tendency and variation matrix for dispersion). We performed ternary plots with the three-movement behaviors to ascertain the distribution of the sample composition. The overlapped heat map allows distinguishing the areas of highest (more intense color) and lowest (less intense color) data concentration. The dispersion structure is represented by 99% and 95% normal-based probability regions around the compositional center. Compositional geometric mean bar plots related to the absolute proportions of time were also generated to display the relative movement behavior profiles for the different frailty status. Finally, compositional data analysis using a multiple linear regression and based on an isometric log-ratio data transformation were performed to define the different associations between movement behaviors and bone health adjusting for time spent in the other behaviors. All statistical analyses were conducted in the three subgroups separately and by sex; nevertheless, since our frail subgroup has a reduced sample size, the results regarding this subgroup were included as Supplementary Materials. The level of statistical significance was set at *p* < 0.05; however, results with *p*-values < 0.06 have been discussed in our study, given that results of medical research should be interpreted in the context of the type of study and other available evidence and, in this case, it could provide important information [29]. Likewise, different variables were considered as covariates to the statistical analysis by backward elimination if the predictor was *p* < 0.2 and the “none” correction was also used for the complete models. Regarding socio-demographic variables, we used age, gender, education, marital status, and income. Anthropometric and body composition variables considered were body mass index, fat mass and lean mass. For lifestyle factors, we included alcohol intake and smoking and finally, as health variables, we used thyroid disease, arthritis and calcium. 

## 3. Results

### 3.1. Descriptive

Characteristics of the participants are shown in Table 1; in addition, more information related to this sample was described previously [25]. Of the 871 eligible subjects with the three stages completed, 740 participants were included in all analyses; 540 robust, 180 pre-frail and 20 frail elderly people. Participants with a metal prosthesis or similar artifacts, insufficient accelerometer wear time data or missing data related to frailty were excluded. Significant differences were found in age between robust and pre-frail and robust and frail groups (*p* < 0.05) and, in BMI between robust and pre-frail groups (*p* < 0.05). No significant differences were found between pre-frail and frail subgroups.

**Figure 1:**
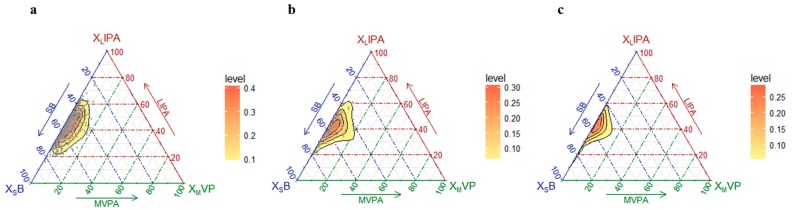
Ternary plots of the sample compositions of time spent in sedentary behavior (SB), light intensity physical activity (LPA) and moderate-to-vigorous intensity physical activity (MVPA) for the robust (**a**), pre-frail (**b**) and frail (**c**) subgroups.

The geometric means for the minutes/day and the % of time spent in each movement behavior are shown in Supplementary Table S1. The variability of the data is summarized in the variation matrix containing all pair-wise log-ratio variances (Supplementary Table S2) and in the matrix of ternary plots with three behaviors represented simultaneously (Figure 1). 

### 3.2. Movement Patterns According to Frailty Status and Sex: Composition of the Day 

**Figure 2:**
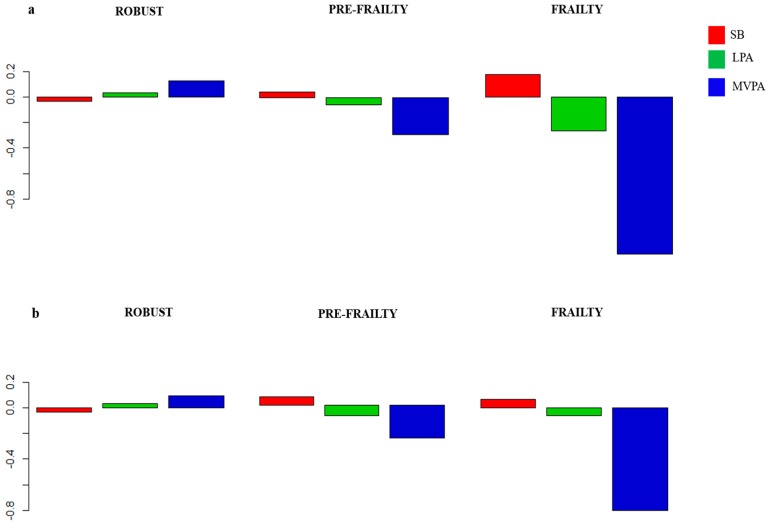
Movement patterns according to frailty status and sex: Compositional geometric mean bar plots comparing the compositional mean of the entire sample with the compositional mean of robust, pre-frail and frail subgroups for sedentary behavior (SB), light intensity physical activity (LPA) and moderate-to-vigorous intensity physical activity (MVPA) for male (**a**) and female (**b**) subgroups.

**Figure 3:**
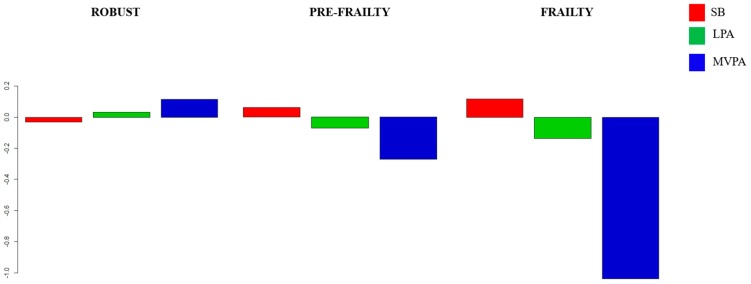
Movement patterns according to frailty status and sex: Compositional geometric mean bar plots comparing the compositional mean of the entire sample with the compositional mean of robust, pre-frail and frail subgroups for sedentary behavior (SB), light intensity physical activity (LPA) and moderate-to-vigorous intensity physical activity (MVPA) for the whole sample.

The composition of the day is presented using compositional mean bar plots for each sex in Figure 2 (for the whole sample see Figure 3). The figure just indicates the movement patterns, of which the effect on bone health will be independently analyzed in the section below. Frail and pre-frail participants spent less time in light intensity PA (LPA) and moderate-to-vigorous intensity PA (MVPA) and more time in SB relative to the entire sample, both in men and women. The opposite tendency was observed in robust older people. 

### 3.3. Compositional Data

The compositional data analysis models by frailty status for the whole sample (without differentiation by sex) are reported in Table 2, as well as in Table 3 for men and Table 4 for women. Likewise, the compositional data analysis models for frail subgroups are reported in Supplementary Table S3. 

### 3.4. Men

The composition of movement behaviors as a whole in the robust subgroup was significantly associated with whole body BMD (*p* = 0.043) and leg BMC and BMD (*p* = 0.054 and *p* = 0.046, respectively). Time spent in SB relative to other movement behaviors was negatively associated with whole body BMD (*p* = 0.041) and leg BMC and BMD (*p* = 0.047 and *p* = 0.036, respectively). No significant associations were found in the pre-frail subgroup. 

### 3.5. Women

The composition of movement behaviors as a whole in the robust subgroup was significantly associated with arm BMC (*p* = 0.015) and whole body and leg BMD and BMC (*p* = 0.033, *p* = 0.000, *p* = 0.008 and *p* = 0.000, respectively). Time spent in SB relative to other movement behaviors was positively associated with whole body and leg BMC and BMD (*p* = 0.020, *p* = 0.000, *p* = 0.003 and *p* = 0.000, respectively). Time spent in LPA was negatively associated with whole body BMD and leg BMC and BMD (*p* = 0.024, *p* = 0.000, and *p* = 0.000, respectively) and positively associated with arm BMC (*p* = 0.011). Time spent in MVPA was negatively associated with arm BMC (*p* = 0.011). Just time spent in MVPA relative to the other movement behaviors was positively associated with leg BMC (*p* = 0.054) and whole body BMC and BMD (*p* = 0.032 and *p* = 0.050, respectively) in pre-frail women. 

## 4. Discussion

To our knowledge, this is the first study to comprehensively analyze the combined effects between the relative distribution of time spent in SB, LPA and MVPA, bone mass and frailty in older adults. The main findings were that despite the lack of sex differences in the movement profiles when comparing frailty status, we found sex differences in the associations of PA and SB on bone mass in the comparisons between frailty groups.

In spite of PA benefits having been extensively demonstrated in the older population as well as the preventive effect of PA for frailty and bone fracture risk [30,31], our study shows some discrepancies. In robust older men, neither the associations between LPA nor MVPA relative to other behaviors and bone mass have been significant, although SB was negatively associated with whole body and leg bone mass. In women, the results were opposite to the men´s group and we even detected a positive relationship between leg and whole body bone mass and SB. In the same way, other studies also found a positive association between SB and bone mass at the femoral neck in older women [25,32,33]. Perhaps the reason for these confusing findings could be the differences in the modality of exercise performed between sex [34,35], as older women practice less osteogenic activities than older men [35]. In addition, even free-living, high-impact PA may not be sufficient to generate increases in BMD in older women [36,37]. Thus, for maintaining bone mass during aging it may be necessary to consider other factors such as the improvement of the geometric response of bone to loading [36,37]. According to SB related to the other movement behaviors, it is possible that the relationship is driven by the ratio between rest and LPA rather than purely the SB time, and if LPA decreases, the relative time in SB increases. Nevertheless, in robust men, SB relative to other movement behaviors was negatively associated with the whole body and leg bone mass, as previously shown in a study on older men [34]. Thus, decrease SB in the way indicated in the models might be a good strategy in this specific population in order to have better bone health. Due to differences in the associations between BMD and PA by sex, these should be studied separately as they appear to be sex dependent [25,35].

Despite the controversial results in robust older women, when women begin to develop frailty, PA seems to be relevant, which may be utilized to improve bone health in this population. Particularly, pre-frail women showed a positive association with bone mass related to MVPA. Cook et al. (2017) also found some evidence of this beneficial effect of PA in those classified as pre-frail compared to robust and frail [9]. Similarly, Yoneki et al. (2018) also recently found that low PA in women and men were negatively associated with bone parameters [3]. The difference in the associations according to PA and SB between robust and pre-frail older women could be because pre-frailty status may represent the sum of several negative conditions and diseases that patients have accumulated over a lifetime. Therefore, pre-frailty status makes older people more sensitive to adverse health outcomes and to the benefits of treatments such as PA [38]. Since pre-frailty is a reversible stage of a potential functional decline and evolution into a disability that could be treated and prevented [39,40], these novel discoveries could indicate an excellent strategy to improve the bone health in older women. Perhaps, we did not find any associations in the men subgroup because it has recently been shown that PA may not have an important effect on the skeletal muscle in pre-frail men [41], which is an excellent indicator of bone mechanical stimulation and its changes are highly correlated with bone health in other populations [42].

In relation to the frail subgroups (men and women), our sample was insufficiently large and therefore, our data does not allow to draw any meaningful conclusions with adequate power. However, due to the differences between different frailty status and the need to study each frailty status independently [10,11], our study did not combine the pre-frail and frail subgroups in order to increase the statistical power. The main reason for that is the increase of comorbidities that occurs with the frailty that could cover up the physical activity effects on pre-frail people [43]. Similarly, the mechanism linking frailty and bone health is likely to be multifactorial being more difficult to differentiate the PA effect if subjects are mixed in the same group [44]. Our data related to the frailty subgroup are included in the Supplementary Materials showing a trend that should be confirmed in future studies including a larger sample in order to explore the benefits of PA on frailty older people and whether it could help to maintain the bone health of this population. 

Finally, in relation to the movement patterns, in our study the frail and pre-frail clearly showed the opposite profile of movement behaviors compared to robust older people, both in the whole sample and also in relation to each sex, spending more time in SB and less in LPA and MVPA than the entire sample, similarly to other previously published studies [30,45,46]. Consequently, older men and women seem to follow similar patterns related to PA and SB, even though the effect of these movement behaviors on bone health differs according to sex and frailty status.

Our study is not without limitations. Despite using older adult-specific cut-off points for vector magnitude counts per minute in this study [27,28], specific cut-off points for frail older people would be necessary. Related to the data, the probability of some results might be attributed to chance due to multiple comparisons cannot be excluded; however, the complete models were corrected and compositional data analyses eliminate collinearity problems and deal with the co-dependence between time spent in different movement behaviors [47,48]. According to the sample, there was a discrepancy between frailty subgroups; however, the distribution of the frailty status prevalence´s is also dissimilar in the general population [19,21]. In addition, due to a large number of variables evaluated in this study, the possibility of obtaining all the data from subjects with worse health status was reduced. And therefore, the sample size of frail people is reduced. The cross-sectional nature of the research design used does not allow definitive conclusions to be drawn around the causal relationship between the variables of the study [19]. Despite these limitations, the principal strength of this study includes having a relatively large cohort of older people in the whole group and the use of a novel statistical analysis (compositional data analysis) for PA behaviors. Moreover, we included some confounder factors as covariates and we assessed body composition and bone mass with the gold standard DXA method, which was requested in a previous compositional study [47]. Finally, we used accelerometers for objectively assessing SB and PA, which is limited in this population [5]. 

## 5. Conclusions

While the whole group, without sex differentiation, does not show a consistent result, the PA effects on bone health are clearer when men and women are studied separately and according to the frailty status. We identified that to combine a decrease of SB and maintenance or increase of LPA and MVPA could produce a beneficial effect on bone mass in robust men. Nonetheless, to increase MVPA and maintain or decrease time spent in LPA and SB, respectively, contributes toward a more favorable bone mass in the case of pre-frail women. In accordance with the movement pattern, pre-frail and frail older people spent more time in SB and less in LPA and MVPA than the entire sample, the opposite movement profile than the robust subgroup. Future research in this area could look at defining guidelines/levels that people should use to increase their bone health depending on their frailty status.

## Figures and Tables

**Table 1:** Anthropometric and descriptive data.

Variables	Whole Sample (*n* = 740)	Robust (*n* = 540)	Pre-frailty (*n* = 180)	Frailty (*n* = 20)
Sex (%)				
Men	46.6	46.5	47.2	45.0
Women	53.4	53.5	52.8	55.0
Age (years)	76.8 ± 4.9	76.0 ± 4.4	78.6 ± 5.5 ☨	80.1 ± 5.1 *
Body mass (kg)	73.6 ± 12.8	73.3 ± 12.5	74.8 ± 13.5	72.3 ± 13.8
Height (cm)	155.9 ± 9.0	156.3 ± 8.9	155.1 ± 9.3	154.4 ± 10.0
BMI (kg/m^2^)	30.3 ± 4.8	30.0 ± 4.7	31.1 ± 4.9 ☨	30.5 ± 5.8
%Body fat	36.6 ± 7.7	36.3 ± 7.7	37.5 ± 7.7	36.4 ± 8.5
Sarcopenia (%)	33.5	37.6	21.3	35.0
Bone health (%)				
Normal bone health	32.3	34.6	25.8	30
Osteopenia	55.1	54.9	55.6	55
Osteoporosis	12.6	10.5	18.6	15
Highest household educational (%)				
Less than primary school graduation	62.3	59.3	68.9	85.0
Primary school graduation	22.4	23.9	18.9	15.0
Secondary school graduation or more	14.6	16.1	11.7	0.0
Marital status (%)				
Single	5.5	5.6	5.6	5.0
Married	70.4	72.2	67.8	45.0
Widower	21.9	19.7	25.6	50.0
Separated/Divorced	1.6	2.0	0.6	0.0

Data are mean ± SD. BMI, body mass index. Statistical significance: *p* < 0.05. * for robust vs. frail older people and, ☨ for robust vs. pre-frail older people.

**Table 2:** Compositional behavior model for bone mass variables for the proportion of the waking hours of the day spent in SB, LPA and MVPA for robust older people (**a**) and pre-frail older people (**b**).

**a.**							
**OUTCOME**	**MODEL *p*-VALUE**	**γ SB**	***p*-VALUE**	**γ LPA**	***p*-VALUE**	**γ MVPA**	***p*-VALUE**
BMC VALUES							
Whole body	0.742	12.497	0.441	−11.938	0.495	−0.559	0.949
Arms (mean)	0.200	−1.961	0.112	*2.391*	*0.073*	−0.429	0.517
Legs (mean)	**0.010**	**9.776**	**0.003**	**−10.420**	**0.004**	0.643	0.717
Lumbar (mean L_1_–L_4_)	0.775	−0.107	0.582	0.148	0.482	−0.041	0.698
Femoral neck	0.732	−0.048	0.461	0.055	0.435	−0.007	0.846
BMD VALUES							
Whole body	0.220	0.010	0.114	−*0.012*	*0.083*	0.002	0.589
Arms (mean)	0.527	−0.004	0.275	0.005	0.273	0.000	0.863
Legs (mean)	**0.000**	**0.035**	**0.000**	**−0.037**	**0.000**	0.003	0.464
Lumbar (mean L_1_–L_4_)	0.749	−0.004	0.664	0.007	0.492	−0.003	0.569
Femoral neck	0.628	−0.007	0.336	0.007	0.386	0.000	0.957
**b.**							
**OUTCOME**	**MODEL *p*-VALUE**	**γ SB**	***p*-VALUE**	**γ LPA**	***p*-VALUE**	**γ MVPA**	***p*-VALUE**
BMC VALUES							
Whole body	0.041	−1.032	0.981	−36.195	0.452	**37.228**	**0.014**
Arms (mean)	0.215	−5.147	0.160	4.216	0.297	0.931	0.434
Legs (mean)	**0.011**	2.717	0.686	−9.971	0.191	**7.253**	**0.003**
Lumbar (mean L_1_–L_4_)	0.794	0.125	0.827	−0.242	0.698	0.118	0.504
Femoral neck	0.136	0.078	0.602	−0.170	0.298	**0.092**	**0.047**
BMD VALUES							
Whole body	**0.033**	0.006	0.704	−0.02	0.250	**0.014**	**0.009**
Arms (mean)	0.872	−0.003	0.747	0.002	0.846	0.001	0.726
Legs (mean)	**0.054**	0.006	0.659	−0.019	0.248	**0.013**	**0.016**
Lumbar (mean L_1_–L_4_)	0.495	0.001	0.975	−0.011	0.719	0.011	0.241
Femoral neck	0.233	0.003	0.902	−0.014	0.557	*0.011*	*0.090*

All models are adjusted for age, gender, education, marital status, income, BMI, fat mass, lean mass, alcohol intake, smoking, thyroid disease, arthritis and calcium, by backward elimination (with predictor retained if *p* < 0.2). Statistically significant associations (*p* < 0.05) are highlighted in bold and the trends are in italics. SB, sedentary behavior; LPA, light intensity physical activity; MVPA, moderate-to-vigorous intensity physical activity; BMC, bone mineral content; BMD, bone mineral density.

**Table 3:** Compositional behavior model for bone mass variables for the proportion of the waking hours of the day spent in SB, LPA and MVPA for robust men (**a**) and pre-frail men subgroup (**b**).

**a.**							
**OUTCOME**	**MODEL *p*-VALUE**	**γ SB**	***p*-VALUE**	**γ LPA**	***p*-VALUE**	**γ MVPA**	***p*-VALUE**
BMC VALUES							
Whole body	0.211	−34.310	0.101	17.100	0.491	17.210	0.340
Arms (mean)	0.357	−2.260	0.155	1.665	0.369	0.595	0.650
Legs (mean)	*0.054*	**−8.067**	**0.047**	2.235	0.638	*5.832*	*0.087*
Lumbar (mean L_1_–L_4_)	0.245	−*0.439*	*0.094*	0.328	0.282	0.111	0.617
Femoral neck	0.987	−0.004	0.957	−0.007	0.945	0.011	0.874
BMD VALUES							
Whole body	**0.043**	**−0.015**	**0.041**	0.003	0.707	*0.012*	*0.064*
Arms (mean)	0.361	−0.006	0.188	0.003	0.571	0.003	0.425
Legs (mean)	**0.046**	**−0.018**	**0.036**	0.006	0.580	*0.012*	*0.084*
Lumbar (mean L_1_–L_4_)	0.326	−0.019	0.136	0.017	0.255	0.002	0.845
Femoral neck	0.210	−*0.016*	*0.078*	0.012	0.241	0.004	0.638
**b.**							
**OUTCOME**	**MODEL *p*-VALUE**	**γ SB**	***p*-VALUE**	**γ LPA**	***p*-VALUE**	**γ MVPA**	***p*-VALUE**
BMC VALUES							
Whole body	0.160	−124.35	0.182	91.65	0.401	32.700	0.362
Arms (mean)	0.467	−3.269	0.574	1.672	0.795	1.597	0.353
Legs (mean)	0.420	7.805	0.518	−13.947	0.335	6.142	0.194
Lumbar (mean L_1_–L_4_)	0.689	−0.599	0.528	0.476	0.654	0.123	0.679
Femoral neck	0.348	−0.061	0.801	−0.041	0.880	0.103	0.191
BMD VALUES							
Whole body	0.246	−0.027	0.275	0.019	0.522	0.008	0.378
Arms (mean)	0.980	−0.003	0.868	0.003	0.897	0.000	0.955
Legs (mean)	0.712	0.000	0.990	−0.007	0.804	0.007	0.450
Lumbar (mean L_1_–L_4_)	0.173	−0.035	0.394	0.017	0.714	0.018	0.157
Femoral neck	0.233	−0.011	0.743	−0.005	0.886	0.016	0.128

All models are adjusted for age, gender, education, marital status, income, BMI, fat mass, lean mass, alcohol intake, smoking, thyroid disease, arthritis and calcium, by backward elimination (with predictor retained if *p* < 0.2). Statistically significant associations (*p* < 0.05) are highlighted in bold and the trends are in italics. SB, sedentary behavior; LPA, light intensity physical activity; MVPA, moderate-to-vigorous intensity physical activity; BMC, bone mineral content; BMD, bone mineral density.

**Table 4:** Compositional behavior model for bone mass variables for the proportion of the waking hours of day spent in SB, LPA and MVPA for robust women (**a**) and pre-frail women subgroup (**b**).

**a.**							
**OUTCOME**	**MODEL *p-*VALUE**	**γ SB**	***p*-VALUE**	**γ LPA**	***p*-VALUE**	**γ MVPA**	***p*-VALUE**
BMC VALUES							
Whole body	**0.033**	**36.761**	**0.020**	−25.831	0.158	−10.93	0.235
Arms (mean)	**0.015**	−1.749	0.154	**3.705**	**0.011**	**−1.954**	**0.011**
Legs (mean)	**0.000**	**19.032**	**0.000**	**−17.361**	**0.000**	−1.672	0.371
Lumbar (mean L_1_–L_4_)	0.205	0.220	0.218	−0.080	0.680	−0.140	0.156
Femoral neck	0.692	−0.046	0.500	0.062	0.395	−0.017	0.661
BMD VALUES							
Whole body	**0.008**	**0.020**	**0.003**	**−0.018**	**0.024**	−0.003	0.494
Arms (mean)	0.209	−0.006	0.147	*0.009*	*0.078*	−0.003	0.295
Legs (mean)	**0.000**	**0.066**	**0.000**	**−0.063**	**0.000**	−0.002	0.648
Lumbar (mean L_1_–L_4_)	0.343	0.011	0.309	−0.004	0.728	−0.007	0.246
Femoral neck	0.292	0.0025	0.787	0.005	0.583	−0.007	0.117
**b.**							
**OUTCOME**	**MODEL *p-*VALUE**	**γ SB**	***p*-VALUE**	**γ LPA**	***p*-VALUE**	**γ MVPA**	***p*-VALUE**
BMC VALUES							
Whole body	*0.097*	38.45	0.546	−79.000	0.260	**40.55**	**0.032**
Arms (mean)	0.192	−4.518	0.230	3.387	0.403	1.131	0.254
Legs (mean)	0.144	7.047	0.446	−12.592	0.219	*5.545*	*0.054*
Lumbar (mean L_1_–L_4_)	0.714	0.291	0.655	−0.188	0.788	−0.17	0.546
Femoral neck	0.546	0.030	0.873	−0.080	0.688	0.050	0.273
BMD VALUES							
Whole body	0.132	0.027	0.286	−0.041	0.140	**0.014**	**0.050**
Arms (mean)	0.550	−0.007	0.614	0.004	0.799	0.003	0.386
Legs (mean)	0.151	0.026	0.249	−0.038	0.125	*0.012*	*0.078*
Lumbar (mean L_1_–L_4_)	0.844	0.013	0.748	−0.009	0.845	−0.005	0.671
Femoral neck	0.222	0.011	0.681	−0.022	0.427	*0.011*	*0.084*

All models are adjusted for age, gender, education, marital status, income, BMI, fat mass, lean mass, alcohol intake, smoking, thyroid disease, arthritis and calcium, by backward elimination (with predictor retained if *p* < 0.2). Statistically significant associations (*p* < 0.05) are highlighted in bold and the trends are in italics. SB, sedentary behavior; LPA, light intensity physical activity; MVPA, moderate-to-vigorous intensity physical activity, BMC, bone mineral content; BMD, bone mineral density.

## Supplementary Materials

The following are available online at https://www.mdpi.com/2072-6643/11/3/582/s1, Table S1: title, Geometric means for SB, LPA and MVPA in minutes/day and percentage of waking hours dividing by frailty status and sex; Table S2: Pair-wise log-ratio matrix for SB, LPA and MVPA by frailty status and sex; Table S3: Compositional behavior model for bone mass variables for the proportion of the waking hours of day spent in SB, LPA and MVPA for frail older people (**a**), frail older men (**b**) and frail older women (**c**).
