# Supplementary material for: The Impact of Movement Behaviors on Bone Health in Elderly with Adequate Nutritional Status: Compositional Data Analysis Depending on the Frailty Status
Source: Nutrients. 2019 Mar 9;11(3):582. doi: 10.3390/nu11030582 (PMC6472191; doi:10.3390/nu11030582)
Supplement: Supplementary file 1 [file nutrients-11-00582-s001.pdf]

Supplementary Table S1. Geometric means for SB, LPA and MVPA in minutes/day and percentage of waking hours dividing by frailty status and sex.

Sample	Robust			Pre-frailty			Frailty		
	**SB**	**LPA**	**MVPA**	**SB**	**LPA**	**MVPA**	**SB**	**LPA**	**MVPA**
Minutes/day									
Whole Sample	439.4	335.6	28.5	472.2	298.6	19.0	496.2	282.4	9.2
Men	462.5	308.6	37.3	479.5	272.4	23.5	541.5	224.0	9.6
Women	419.4	359.0	20.8	465.7	322.2	15.0	459.0	330.1	8.9
% of waking hours									
Whole Sample	54.8	41.7	3.5	60.0	37.6	2.4	63.7	35.2	1.1
Men	57.4	38.1	4.5	62.1	34.9	3.0	70.6	28.2	1.2
Women	52.6	44.8	2.6	58.2	40.0	1.8	58.0	41.0	1.0

SB, sedentary behavior; LPA, light intensity physical activity; MVPA, moderate-to-vigorous intensity physical activity.

Supplementary Table S2. Pair-wise log-ratio matrix for SB, LPA and MVPA by frailty status and sex.

Sample	Robust older people			Pre-frailty older people			Frailty older people		
	**SB**	**LPA**	**MPA**	**SB**	**LPA**	**MVPA**	**SB**	**LPA**	**MVPA**
Whole sample									
SB	0	0.119	1.195	0	0.203	1.398	0	0.258	1.763
LPA	0.119	0	1.076	0.203	0	1.195	0.258	0	1.505
MVPA	1.195	1.076	0	1.398	1.195	0	1.763	1.505	0
Men									
SB	0	0.178	1.106	0	0.250	1.316	0	0.399	1.770
LPA	0.178	0	0.928	0.250	0	1.066	0.399	0	1.371
MVPA	1.106	0.928	0	1.316	1.066	0	1.770	1.371	0
Women									
SB	0	0.070	1.306	0	0.163	1.510	0	0.151	1.763
LPA	0.070	0	1.236	0.163	0	1.347	0.151	0	1.613
MVPA	1.306	1.236	0	1.510	1.347	0	1.763	1.613	0

SB, sedentary behavior; LPA, light intensity physical activity; MVPA, moderate-to-vigorous intensity physical activity. Note: A value close to zero implies that the times spent in the two behaviors involved in the ratio (arranged by rows and columns) are highly proportional.

Supplementary Table S3. Compositional behavior model for bone mass variables for the proportion of the waking hours of day spent in SB, LPA and MVPA for frail older people (**a**), frail older men (**b**) and frail older women (**c**).

a.

OUTCOME	MODEL *P*-VALUE	γ SB	*P*-VALUE	γ LPA	*P*-VALUE	γ MVPA	*P*-VALUE
			BMC VALUES				

Whole body	0.797	0.681	0.988	-15.421	0.774	14.740	0.514
Arms (mean)	0.306	-17.151	0.164	15.217	0.264	1.934	0.667
Legs (mean)	0.714	-14.804	0.491	19.676	0.425	-4.872	0.606
Lumbar (mean L_1–L_4)	0.125	1.601	0.173	-0.979	0.449	-0.622	0.172
Femoral neck	0.356	-0.384	0.366	0.582	0.225	-0.198	0.219
BMD VALUES							
Whole body	0.392	0.015	0.378	-0.025	0.224	0.010	0.241
Arms (mean)	0.813	-0.004	0.846	0.008	0.695	-0.005	0.538
Legs (mean)	0.843	0.003	0.955	0.009	0.867	-0.012	0.575
Lumbar (mean L_1–L_4)	0.222	0.015	0.812	0.027	0.706	-0.043	0.102
Femoral neck	0.713	-0.014	0.486	0.019	0.431	-0.004	0.590

b.

OUTCOME	MODEL *P*-VALUE	γ SB	*P*-VALUE	γ LPA	*P*-VALUE	γ MVPA	*P*-VALUE
BMC VALUES							
Whole body	0.710	-165.97	0.434	214.950	0.443	-48.97	0.664
Arms (mean)	0.906	-7.404	0.828	3.278	0.940	4.126	0.807
Legs (mean)	0.849	-7.624	0.894	-4.692	0.949	12.316	0.666
Lumbar (mean L_1–L_4)	0.551	0.966	0.626	-0.218	0.931	-0.748	0.473
Femoral neck	0.506	-0.780	0.264	0.973	0.278	-0.193	0.560
BMD VALUES							
Whole body	0.411	-0.120	0.204	0.150	0.224	-0.030	0.522
Arms (mean)	0.721	-0.054	0.471	0.055	0.563	-0.001	0.971
Legs (mean)	0.493	-0.161	0.269	0.174	0.344	-0.014	0.839
Lumbar (mean L_1–L_4)	0.900	-0.009	0.95	0.046	0.818	-0.037	0.670
Femoral neck	0.425	-0.090	0.249	0.127	0.210	-0.037	0.325

c.

OUTCOME	MODEL *P*-VALUE	γ SB	*P*-VALUE	γ LPA	*P*-VALUE	γ MVPA	*P*-VALUE
BMC VALUES							
Whole body	0.121	*-437.74*	*0.054*	**486.210**	**0.050**	-48.48	0.191
Arms (mean)	0.474	-15.826	0.266	18.088	0.246	-2.262	0.392
Legs (mean)	**0.014**	**-88.044**	**0.014**	**105.066**	**0.008**	**-17.021**	**0.019**
Lumbar (mean L_1–L_4)	0.983	0.343	0.871	-0.398	0.863	0.055	0.891
Femoral neck	0.164	-0.934	0.463	1.423	0.313	*-0.489*	*0.065*

BMD VALUES							
Whole body	**0.051**	-0.124	0.172	*0.169*	*0.094*	**-0.045**	**0.020**
Arms (mean)	0.226	0.034	0.340	-0.027	0.476	-0.007	0.320
Legs (mean)	**0.010**	-0.078	0.299	*0.142*	*0.093*	**-0.064**	**0.003**
Lumbar (mean L_1–L_4)	0.552	-0.162	0.295	0.179	0.290	-0.016	0.567
Femoral neck	**0.015**	**-0.303**	**0.005**	**0.336**	**0.005**	*-0.032*	*0.064*

All models are adjusted for age, gender, education, marital status, income, BMI, fat mass, lean mass, alcohol intake, smoking, thyroid disease, arthritis and calcium, by backward elimination (with predictor retained if $p < 0.2$). Statistically significant associations ($p < 0.05$) are highlighted in bold and the trends are in italics. SB, sedentary behavior; LPA, light intensity physical activity; MVPA, moderate-to-vigorous intensity physical activity; BMC, bone mineral content; BMD, bone mineral density.
